# Discrepancies between cardiovascular magnetic resonance and Doppler echocardiography in the measurement of transvalvular gradient in aortic stenosis: the effect of flow vorticity

**DOI:** 10.1186/1532-429X-15-84

**Published:** 2013-09-20

**Authors:** Julio Garcia, Romain Capoulade, Florent Le Ven, Emmanuel Gaillard, Lyes Kadem, Philippe Pibarot, Éric Larose

**Affiliations:** 1 Québec Heart and Lung Institute, Laval University, Québec, Canada; 2 Laboratory of Cardiovascular Fluid Dynamics, Concordia University, Montréal, Canada; 3 Department of Radiology, Northwestern University, Chicago, USA; 4 Department of Mechanical Engineering, McGill University, Montréal, Canada

**Keywords:** Aortic stenosis, Echo-Doppler, Cardiovascular magnetic resonance, Mean pressure gradient, Flow vorticity

## Abstract

**Background:**

Valve effective orifice area EOA and transvalvular mean pressure gradient (MPG) are the most frequently used parameters to assess aortic stenosis (AS) severity. However, MPG measured by cardiovascular magnetic resonance (CMR) may differ from the one measured by transthoracic Doppler-echocardiography (TTE). The objectives of this study were: 1) to identify the factors responsible for the MPG measurement discrepancies by CMR versus TTE in AS patients; 2) to investigate the effect of flow vorticity on AS severity assessment by CMR; and 3) to evaluate two models reconciling MPG discrepancies between CMR/TTE measurements.

**Methods:**

Eight healthy subjects and 60 patients with AS underwent TTE and CMR. Strouhal number (St), energy loss (EL), and vorticity were computed from CMR. Two correction models were evaluated: 1) based on the Gorlin equation (MPG_CMR-Gorlin_); 2) based on a multivariate regression model (MPG_CMR-Predicted_).

**Results:**

MPG_CMR_ underestimated MPG_TTE_ (bias = −6.5 mmHg, limits of agreement from −18.3 to 5.2 mmHg). On multivariate regression analysis, St (p = 0.002), EL (p = 0.001), and mean systolic vorticity (p < 0.001) were independently associated with larger MPG discrepancies between CMR and TTE. MPG_CMR-Gorlin_ and MPG_TTE_ correlation and agreement were r = 0.7; bias = −2.8 mmHg, limits of agreement from −18.4 to 12.9 mmHg. MPG_CMR-Predicted_ model showed better correlation and agreement with MPG_TTE_ (r = 0.82; bias = 0.5 mmHg, limits of agreement from −9.1 to 10.2 mmHg) than measured MPG_CMR_ and MPG_CMR-Gorlin_.

**Conclusion:**

Flow vorticity is one of the main factors responsible for MPG discrepancies between CMR and TTE.

## Background

Valve effective orifice area (EOA) and mean transvalvular pressure gradient (MPG) are the most frequently used parameters to assess aortic stenosis (AS) severity [1]. Current ACC/AHA and ESC guidelines suggest an EOA < 1.0 cm^2^ and a MPG > 40 mmHg as main criteria to define a severe AS [1,2]. Transthoracic Doppler-echocardiography (TTE) is the primary method utilized in clinical practice to assess and grade AS severity. Since TTE has some theoretical and technical limitations [1] cardiovascular magnetic resonance (CMR) has emerged as a non-invasive, radiation-free accurate alternative method to corroborate AS severity when uncertain or discordant results are obtained at TTE [3-6]. However, previous studies have showed that MPG measured by CMR may differ from the one obtained by TTE, mainly when transvalvular velocity is greater than 4 m/s [3,4,7-10]. It was hypothesized that this underestimation might be due to the following factors: flow turbulence generated downstream the severe AS, local signal loss, background noise and phase wrap [7,11-14].

**Figure 1:**
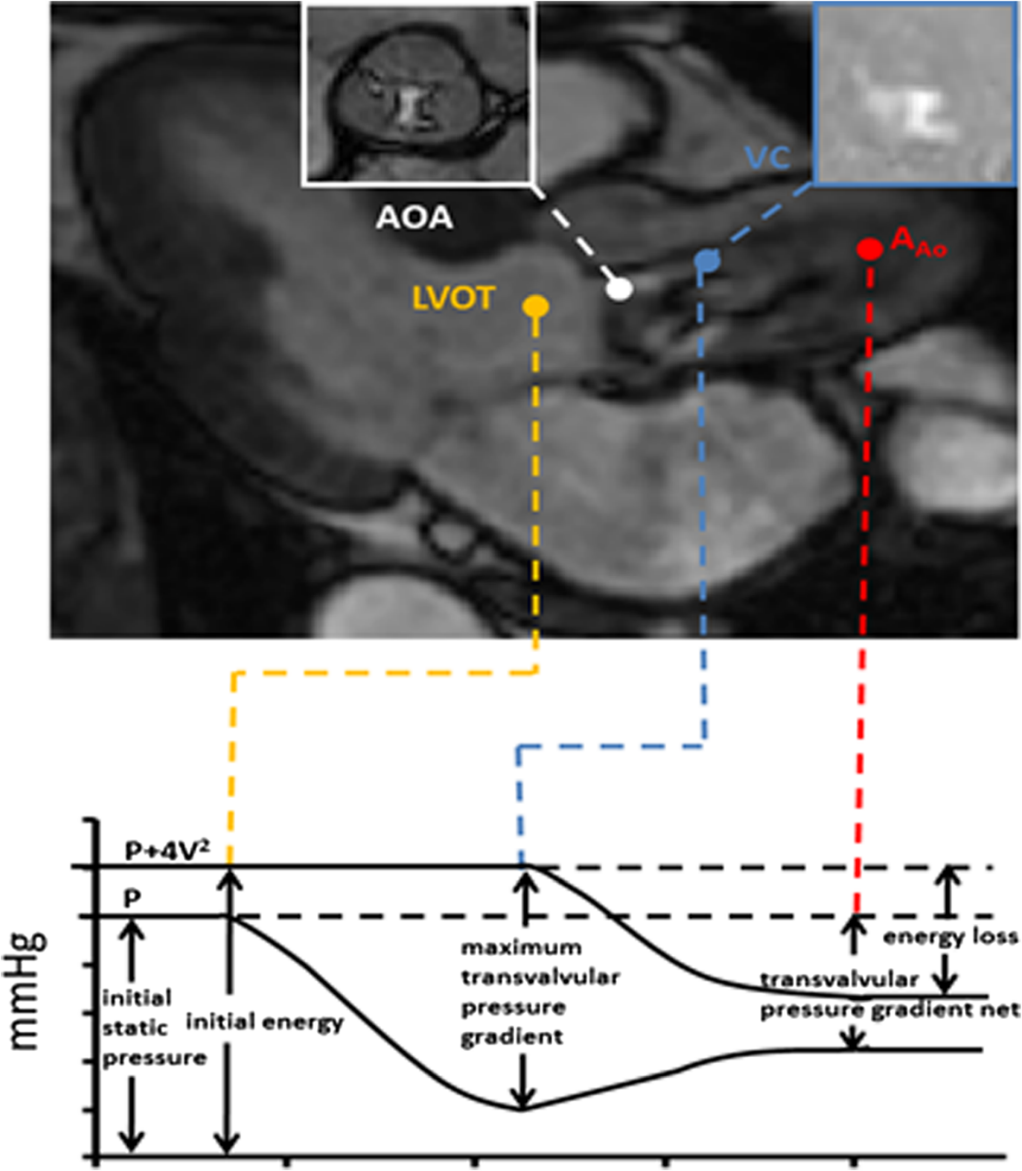
**Fluid mechanics of the aortic valve.** Schematic representation of the system composed of left ventricle, aortic valve and ascending aorta with corresponding static pressure (P) and energy in terms of total pressure (P+4 V^2^). LVOT indicates left ventricular outflow tract, V indicates LVOT velocity, AOA indicates anatomic aortic area and VC indicates the vena contracta position, cross-sectional area of VC corresponds to the valve effective orifice area. Magnetic resonance velocity measurements at vena contracta (10 mm from the aortic valve) were used to compute dimensionless flow parameters and vorticity magnitude. A_Ao_ indicates ascending aorta.

Based on a recent study on the inconsistent grading of AS severity assessed by TTE and cardiac catheterization [15] it has been proposed to use Gorlin equation to correct the MPG disagreement between TTE and CMR measurements [16] leading to reasonable results in the evaluated population. From a fluid dynamic point-of-view, when the blood flows through the aortic valve it is spatially accelerated from the left ventricular outflow tract (LVOT) to the location of the vena contracta and it is then decelerated and diverges within the ascending aorta (Figure 1). This flow generates turbulence when the aortic valve is severely stenotic and an irreversible heat dissipation process. Several flow-derived parameters (energy loss, vorticity, and Strouhal number) may provide an insight on the presence and intensity of turbulence generated downstream of a severe AS [17]. All these flow parameters may be useful for identifying potential sources of discordance between MPG measured by CMR and TTE.

Energy loss (EL) represents the energetic cost (in mmHg) between the LVOT and the ascending aorta after pressure recovery [18-22]. Vorticity intensity (ω) can be used to estimate the dissipation effects within the flow [23]. Interestingly, a recent study demonstrated that vorticity jet shear layer can also be used to estimate EOA using CMR velocity measurements [5]. Strouhal number (St) represents the dimensionless oscillating flow through the aortic valve [24].

The objectives of this study were: 1- to identify the factors responsible for the discrepancies in the MPG measurement by CMR versus TTE in patients with AS, 2- to investigate the effect of vorticity on AS severity assessment by CMR and 3- to evaluate two models to reconciling MPG discrepancies between CMR/TTE measurements.

## Methods

### Study population

Eight (8) healthy control subjects and 60 patients with mild to severe AS (0.60 cm^2^ ≤ EOA_TTE_ ≤ 1.79 cm^2^) underwent comprehensive research TTE and CMR exams in the context of this study. All subjects were prospectively recruited based on standard clinical TTE exams. Both tricuspid and bicuspid AS patients were included. Exclusion criteria were: age < 21 years old, LV ejection fraction < 50%, more than mild mitral disease or aortic regurgitation, poor TTE imaging quality and standard contra-indications to magnetic resonance imaging. The study was approved by the Ethical Review Board (Comité d’Éthique de la Recherche, Institut Universitaire de Cardiologie et de Pneumologie de Québec) and all patients provided written informed consent for their participation in this study.

### Transthoracic Doppler-echocardiography

TTE studies were performed and analyzed by two experienced echocardiographers according to the American Society of Echocardiography guidelines [25] and included:

1) Valve hemodynamics: transvalvular pressure gradients were determined by simplified Bernoulli formula: MPG_TTE_ = 4 × V_mean_^2^, where V is the mean systolic aortic transvalvular velocity; and valve EOA was calculated by continuity equation: EOA_TTE_ =  SV_LVOT_/ VTI_Ao_ =  (VTI_LVOT_ ×  A_LVOT_) / VTI_Ao__,_ where SV_LVOT_ is the stroke volume measured in the LVOT, A_LVOT_ is the cross-sectional area of the LVOT and VTI_LVOT_ and VTI_Ao_ are the velocity-time integrals at the LVOT and the vena contracta, respectively. AS severity was classified on the basis of TTE-derived EOA: Mild to moderate (EOA> 1.0 cm^2^) and severe (EOA ≤ 1.0 cm^2^);

2) Parameters of arterial hemodynamics: Systemic arterial compliance (SAC) was computed using the following formula: SAC = SV_i_/PP, where SV_i_ is the stroke volume indexed to the body surface area and PP is the pulse arterial pressure. Systemic vascular resistance (SVR) was also estimated from the following formula: SVR  = 80MAP/80  ×  MAP /CO, where MAP is the mean arterial pressure and CO is the cardiac output.

### Cardiovascular magnetic resonance

CMR studies were performed 2 to 4 weeks after TTE with patients in comparable hemodynamic state (SV_TTE_ = 80±14 vs. SV_CMR_ = 77±17, p=NS; heart rate TTE = 63±10 bpm vs. heart rate CMR = 65±11 bpm, p = NS). Imaging was performed with a 1.5 Tesla Philips Achieva scanner operating release 2.6 level 3 and dedicated phased-array cardiac coil during successive end-expiratory breath-holds (Philips Healthcare, Best, The Netherlands) as described in previous studies [3,5,6]. Typical parameters included TR/TE of 3.4/1.2 ms, flip angle 40°, NEX of 1, yielding in-plane spatial resolution of 1.6×2 mm. Through-plane phase-contrast imaging was performed during breath-hold in the LVOT at 12 mm upstream from the aortic valve annulus (reference: 0 mm), in the ascending aorta (Ao) at 10 mm downstream of the aortic annulus, both planes parallel to the aortic valve annulus plane. Flow imaging parameters consisted of: TR/TE = 4.60-4.92/2.76-3.05 ms, flip angle = 15°, 24 phases, pixel spacing = 1.32–2.07 mm, slice thickness = 10 mm and acquisition matrix = 256 × 208, scan time = 10–25 s without SENSE. For each patient, peak aortic jet velocity measured by TTE was used to define CMR encoding velocity (CMR encoding velocity = (1.25 to 1.5) × peak jet velocity, range from 1.5 to 5.5 m/s) to optimally define resolution and avoid signal wrap.

CMR images acquisitions and analyses were performed by investigators blinded to clinical and TTE results. A custom-made research application was developed using Matlab software (Mathworks, Natick, Ma, USA) to process and analyze velocity-encoded images [5,26] and the image stack was processed to filter background noise. Regions of interest (ROIs) were defined on each of the 24 phases of anatomical magnitude images to include the lumen of the LVOT and of the aorta. The following measurements were performed within each ROI on matched phase images at LVOT and Ao positions.

The peak and average flow velocities within the ROI were used to determine the changes in instantaneous peak and average velocity (V_average_) in the LVOT during the cardiac cycle. The instantaneous LVOT flow rate was calculated by multiplying the instantaneous V_average_ by the LVOT cross-sectional area.

The maximum through-plane flow velocity within the ROI was used to determine the instantaneous peak aortic velocity at Ao position. The mean transvalvular velocity was computed using instantaneous peak velocity values during systole (i.e. ejection period).

### CMR valve hemodynamic parameters

Mean transvalvular pressure gradient (MGP_CMR_) was determined by simplified Bernoulli formula and valve EOA was calculated using jet shear layer detection method [5] from velocity field at Ao plane (i.e. 10 mm downstream of the aortic annulus). The same plane was used to compute energy loss (EL = V_peak_^2^ × [1 − EOA_CMR_/A_Ao_]^2^), where V_peak_^2^ is the systolic transvalvular aortic peak jet velocity and A_Ao_ is cross-sectional area of the ascending aorta. Systolic mean vorticity ($\omega \left[t\right]=\frac{\partial V\left[t\right]}{\partial x}-\frac{\partial V\left[t\right]}{\partial y}$) was computed using a compact-Richardson interpolation scheme [27-29], absolute value was used to consider both clockwise and anti-clockwise effects. Briefly, compact-Richardson interpolation scheme estimates the partial velocity derivatives needed for the vorticity computation with reduced partial volume effects and local noise alterations using an iterative-weighted process. This vorticity method works with through-plane (single velocity component) and full volume (three velocity components, i.e. full vector) velocity data, and it has been previously validated in silico, in vitro and in vivo [27-29]. Furthermore Strouhal number (non-dimensional oscillating flow) was given by St  =  (D_average_//2)  ×  (*f*/[/ [V_peak_ −  V_average_]), where D_average_ is the averaged systolic diameter of LVOT and *f* is the heart rate.

To assess the discordance between MPG obtained by CMR and MPG obtained by TTE, the MPG relative error (in %) was computed as follows: MPG_error_ =  ([MPG_TTE_  −  MPG_CMR_]/] /MPG_TTE_)  ×  100. Absolute error differences (|∆MPG|) were classified in three groups: group A (|∆MPG| ≤ 10 mmHg), group B (10 mmHg < |∆MPG| < 20 mmHg) and group C (|∆MPG| ≥ 20 mmHg). Predicted MPG_CMR-Gorlin_ was computed using Gorlin equation as follows: MPG_CMR − Gorlin_ =  (CO/[/ [HR  ×  SEP  ×  44.3  ×  EOA_CMR_])^2^[15,16,30], where CO is the cardiac output, HR is the heart rate and SEP is the systolic ejection period.

### Statistical analyses

Results are expressed as mean ± SD. Comparisons between groups (healthy control subjects vs. moderate vs. severe AS or tricuspid vs. bicuspid valve) were performed with the use of Student *t*-tests or One-way ANOVA when appropriate. Association and agreement between variables were assessed by Pearson’s correlations and Bland-Altman methods, respectively. Multivariate linear regression analysis was performed to identify the factors independently associated with MPG_error_ and MPG_TTE_. We included in multivariate models age and AS severity defined by CMR (i.e. EOA_CMR_ or MPG_CMR_) and all variables with p-value<0.15 in univariate analysis. Standardized regression coefficients were presented as mean ± standard error (βeta coeff ± SE). Statistical analysis was performed with SPSS 17 (SPSS, Chicago, IL).

## Results

Sixty patients with mild to severe AS (65% men, age 64±15 years) and eight healthy subjects (75% men, age 34±8 years) were included in this study. The demographic, TTE and CMR data of the patients with AS and the healthy subjects are presented in Table 1. Valve morphology was bicuspid in 27% of AS patients. Age, MPG, EL, vorticity, and Strouhal number were significantly higher in AS patients compared with healthy control subjects. When comparing AS severity groups with healthy control subjects a significant difference was found for AS severity indices, EL, vorticity, and Strouhal number. There was a significant difference (p<0.05) between aortic valve phenotype (i.e. bicuspid vs. tricuspid aortic valves) for age, systolic arterial pressure, and systemic compliance.

**Table 1:** Comparison of clinical TTE and CMR data

	**Healthy subjects**	**AS patients**
	**(n=8, mean±SD)**	**(n=60, mean±SD)**
**Patient demographics**		
Age (years)	34 ± 8	64 ± 15 *
Gender (men %)	75	65
Body surface area (m^2^)	1.93 ± 0.26	1.82 ± 0.19
Systolic arterial pressure (mmHg)	116 ± 10	132 ± 23
Diastolic arterial pressure (mmHg)	77 ± 5	72 ± 12
**Doppler echocardiography data**		
Valve phenotype (bicuspid, %)		36
**Aortic valve hemodynamics**		
Stroke volume (mL)	80 ± 20	80 ± 13
Mean transvalvular gradient (mmHg)	5 ± 1	20 ± 10 *
Valve effective orifice area (cm^2^)	2.67 ± 0.47	1.19 ± 0.28 *
**Systemic arterial hemodynamics**		
Systemic arterial compliance (mL.m^-2^.mmHg^-1^)	1.06 ± 0.21	0.91 ± 0.32
Systemic vascular resistance (dyne.s.cm^-5^)	1448 ± 319	1515 ± 338
**Cardiovascular magnetic resonance data**		
**Aortic valve hemodynamics**		
Stroke volume (mL)	84 ± 14	76 ± 17
Mean transvalvular gradient (mmHg)	3 ± 1	12 ± 7 *
Valve effective orifice area (cm^2^)	3.08 ± 0.8	1.4 ± 0.41 *
Energy loss (mmHg)	3.33 ± 1.11	13.81 ± 7.99 *
Mean systolic vorticity (1/s)	88 ± 13	125 ± 35 *
Strouhal	0.0174 ± 0.0034	0.0087 ± 0.0029 *

*:p<0.001 with healthy.

### Factors of the MPG underestimation by CMR

**Figure 2:**
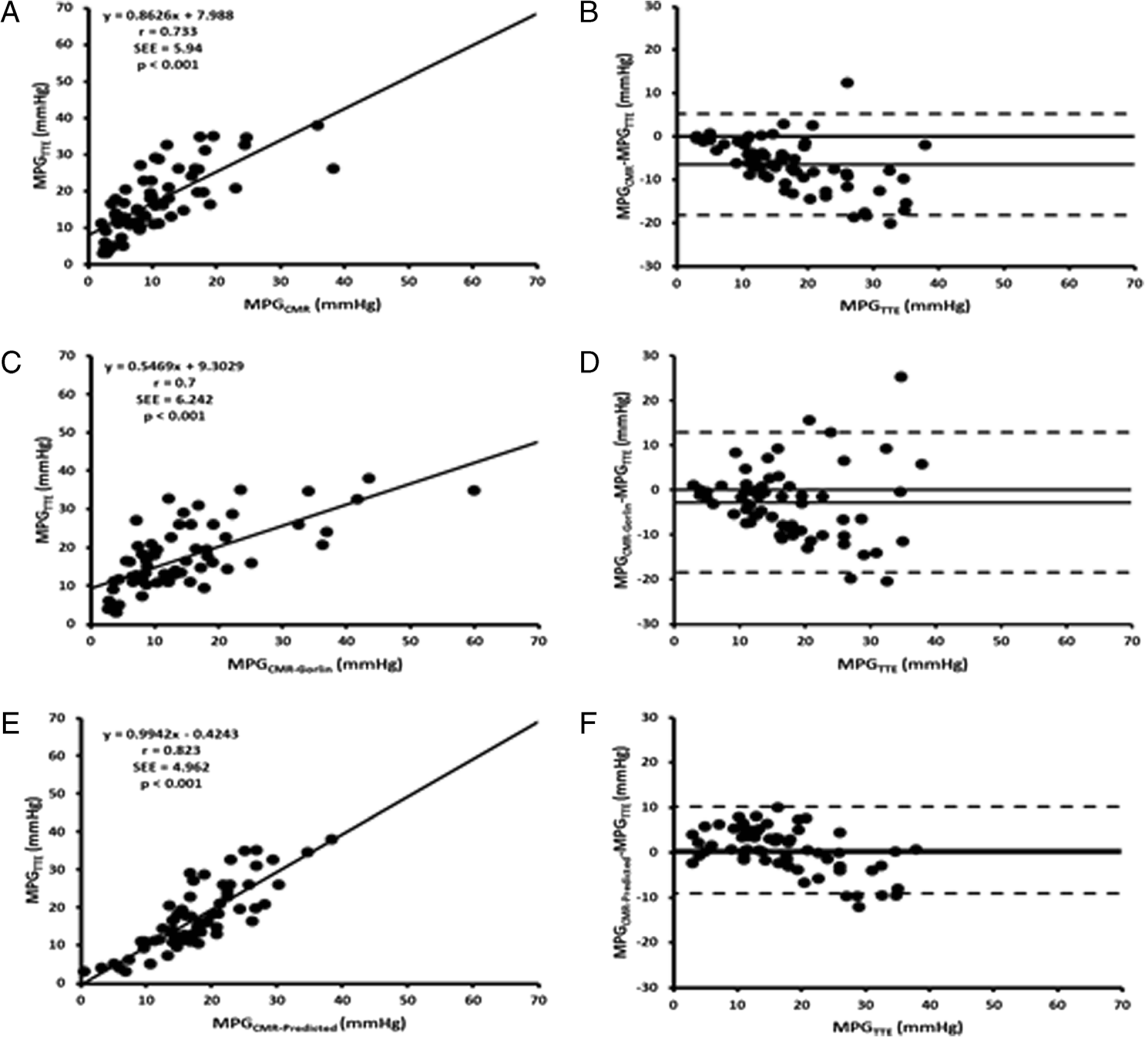
**Comparison of mean transvalvular pressure gradients measured by TTE versus by CMR.** Panel **A** shows the Pearson correlation plot for mean transvalvular pressure gradient measured by TTE (MPG_TTE_) and CMR (MPG_CMR_). Panel **B** shows the corresponding Bland-Altman plot. Panel **C** shows the Pearson correlation plot for mean transvalvular pressure gradient measured by TTE (MPG_TTE_) and predicted by Gorlin equation using CMR (MPG_CMR-Gorlin_). Panel **D** shows the corresponding Bland-Altman plot. Panel **E** shows the Pearson correlation plot for mean transvalvular pressure gradient measured by TTE (MPG_TTE_) and predicted model using vorticity and dimensionless stroke volume from CMR (MPG_CMR-Predicted_). Panel **F** shows the corresponding Bland-Altman plot.

MPG_CMR_ underestimated MPG_TTE_ and this underestimation increased with AS severity (r = 0.73 [Figure 2A]; bias = −6.5 mmHg, limits of agreement from −18.3 to 5.2 mmHg, [Figure 2B], Table 1). Comparison between MPG_CMR_ and MPG_TTE_ was significantly different for all categories (p<0.001). When considering |∆MPG| groups, group A had 78% (n = 53) of subjects, group B had 21% (n = 14) of subjects and group Chad 1% (n = 1) of subjects. Vorticity (p = 0.003), EL (p = 0.006) and Strouhal number (p = 0.123) were significantly related to MPG_error_ (Table 2). In the multivariate analysis, after adjustment for EOA_CMR_ and age, EL, Strouhal number and vorticity were the factors independently associated with higher MPG_error_ (Table 2).

**Table 2:** Univariate and multivariate determinants of relative error in transvalvular mean pressure gradient

**Mean transvalvular pressure gradient**	**Univariate analysis**		**Multivariate model**	
**Relative error = MRI-TTE/TTE (%)**	**βeta coeff ± SE**	**p-value**	**βeta coeff ± SE**	**p-value**
Age (years)	0.19 ± 0.19	0.123	-	0.609
Effective orifice area (cm^2^)*	−0.14 ± 4.66	0.256	-	0.112
Strouhal number*	−0.19 ± 809	0.123	−0.46 ± 934	0.002
Energy loss (mmHg)*	−0.33 ± 0.37	0.006	−0.41 ± 0.36	0.001
Mean systolic vorticity (1/s)*	−0.36 ± 0.09	0.003	−0.53 ± 0.08	<0.001

**Legend:** *Parameters computed from CMR. Multivariate model includes only variables that were significantly (p<0.15) associated with transvalvular mean pressure gradient relative error on univariate analysis. Βeta coeff ± SE were standardized regression coefficients ± standard error.

### TTE and CMR transvalvular mean pressure gradient prediction models

MPG_CMR-Gorlin_ and MPG_TTE_ measurements showed a good correlation and agreement, a reduced underestimation with higher limits of agreement than measured MPG_CMR_ (r = 0.7 [Figure 2C]; bias = −2.8 mmHg, limits of agreement from −18.4 to 12.9 mmHg, [Figure 2D]). When considering |∆MPG| groups, group A had 76% (n = 52) of subjects, group B had 21% (n = 14) of subjects and group C had 3% (n = 2) of subjects.

Independent factors associated to MPG_error_ were included, avoiding redundancies, in a multivariate analysis for MPG_TTE_ adjusted to MPG_CMR_; all the parameters included were significantly associated in the univariate analysis (p < 0.001, see Table 3).

**Table 3:** Univariate and multivariate determinants of Doppler-echocardiography mean transvalvular pressure gradient

**Doppler-Echocardiography mean transvalvular mean pressure gradient (mmHg)**	**Univariate analysis**		**Multivariate model**	
	**βeta coeff ± SE**	**p-value**	**βeta coeff ± SE**	**p-value**
Mean transvalvular pressure gradient (mmHg)*	0.73 ± 0.1	<0.001	0.72 ± 0.19	<0.001
Energy loss (mmHg)*	0.68 ± 0.09	<0.001	-	0.98
Strouhal number*	−0.57 ± 224	<0.001	−0.44 ± 202	<0.001
Mean systolic vorticity*	0.48 ± 0.03	<0.001	−0.21 ± 0.03	0.07

**Legend:** *Parameters measured by CMR. Multivariate model includes only variables that were significantly (p < 0.001) associated with echo-Doppler transvalvular mean pressure gradient on univariate analysis. Βeta coeff ± SE were standardized regression coefficients ± standard error.

In this multivariate analysis MPG_CMR_, Strouhal number and mean vorticity were independently associated to MPG_TTE_. We have introduced a new MPG_CMR-Predicted_ model based in the previous multivariate analysis presented in Table 3:

$$\begin{array}{l} \mathrm{MP}G_{\mathrm{CMR}-\mathrm{Predicted}}=24-0.05\times \mathrm{mean}\;\omega +0.85\\ \;\times \mathrm{MP}G_{\mathrm{CMR}}-960\times \mathrm{St} \end{array}$$

Where ω is the vorticity magnitude and St is the Strouhal number. MPG_CMR-Predicted_ model showed better correlation with MPG_TTE_ and a low overestimation with lower limits of agreement (r = 0.82 [Figure 2E]; bias = 0.5 mmHg, limits of agreement from −9.1 to 10.2 mmHg, [Figure 2F]) than measured MPG_CMR_ and MPG_CMR-Gorlin_. When considering |∆MPG| groups, group A had 99% (n = 67) of subjects and group B had 1% (n = 1) of subjects.

## Discussion

The main findings of this study are: 1) Mean systolic vorticity and EL were the factors associated with the discrepancies between CMR and TTE for the measurement of MPG. 2) Flow vorticity may be used as a quantitative parameter of AS hemodynamic severity; 3) The introduction of a new MPG_CMR-Predicted_ model based on mean vorticity and oscillating flow(Strouhal number) allowing a better correlation and agreement than MPG_CMR-Gorlin_.

MPG_TTE_ underestimation by CMR is typically related to local signal loss, background noise, phase wrap and turbulence [7,9-14,31,32]. However, as it was demonstrated with catheterization other hemodynamic parameters affect mean transvalvular pressure gradients measurements, mainly energy loss [21] and pressure recovery [18-22,33]. Those explanations should also apply to CMR given the theoretical background of the measurements. Some previous studies used those similarities to estimate EOA with CMR [9,10]. Reynolds number (ratio of the inertial/viscous forces) has been shown to be a dimensionless parameter contributing to the explanation of pressure gradient differences between TTE and catheterization in AS [34,35]. However, it is mainly valid in steady flow conditions evaluating flow regimes (laminar or turbulent).

In this study, a predictive MPG model using Gorlin equation [15,16,30] and CMR measurements was used. MPG_CMR-Gorlin_ reduced CMR-TTE bias differences but it showed important differences with AS severity increase (i.e. higher MPG) (Figure 2, panel C and D). It is important to notice that we used the Gorlin equation based on Minners et al. study [15] and preliminary reported results [16]. However, it was been demonstrated that Gorlin equation have two small errors: 1) the use of mean flow instead of root-mean-square flow and 2) the use of the coefficient 44.3 instead of 50.5 [36]. The original intent of Gorlin equation was to give an estimate of anatomic valve area (AVA) instead of EOA. Of interest, EOA represent better the aortic valve hemodynamic than AVA and similar AVA geometries could lead to different EOA, i.e. different AS severities [37].

**Figure 3:**
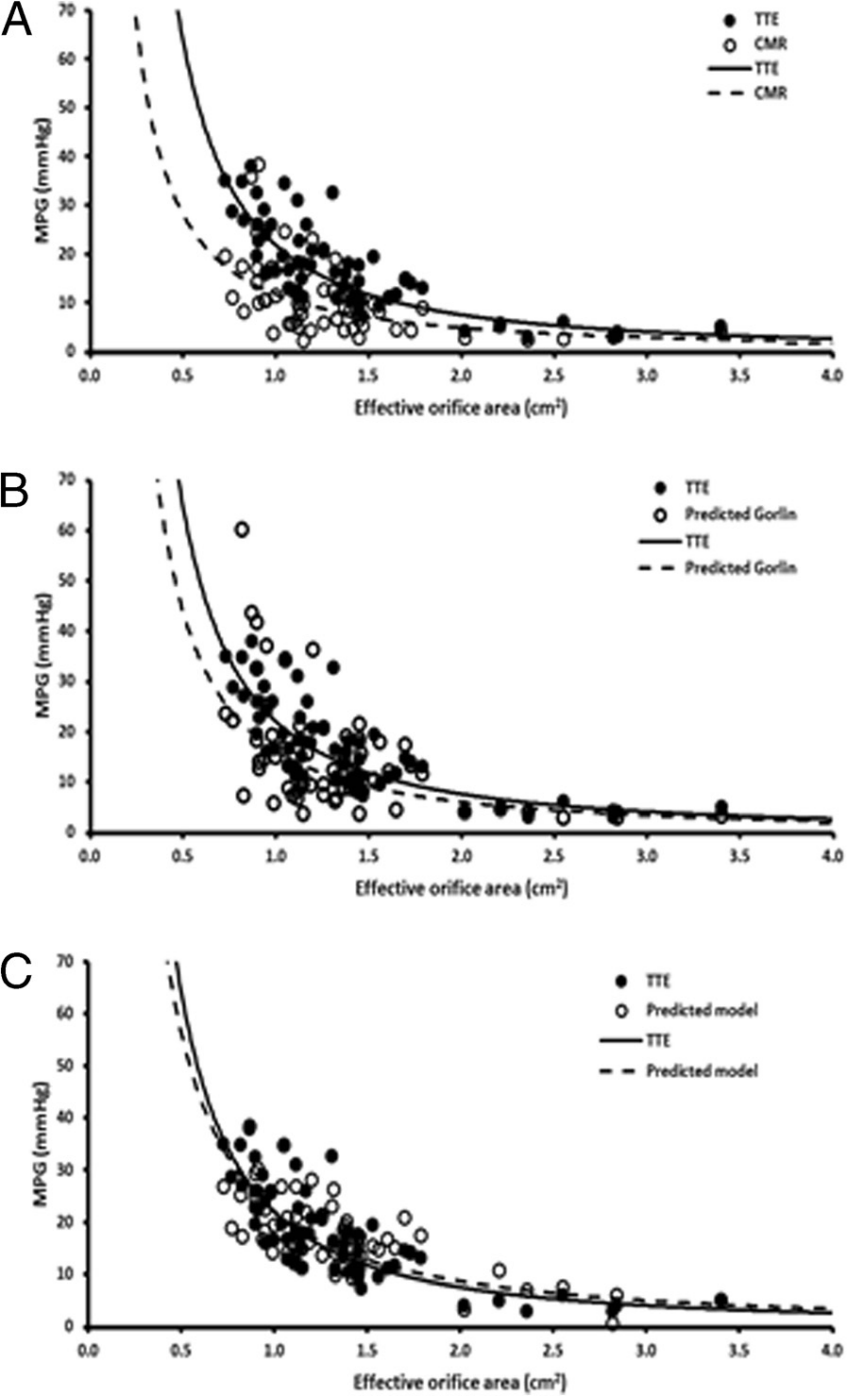
**Effective orifice area and mean transvalvular pressure gradients.** Panel **A** shows the aortic valve effective orifice area and mean transvalvular pressure gradient (MPG) plot using measurements from TTE (MPG_TTE_) and CMR (MPG_CMR_). Panel **B** shows the same plot but using MPG predicted by Gorlin equation and CMR measurements (MPG_CMR-Gorlin_). Panel **C** shows the same plot but using MPG predicted using mean vorticity magnitude and dimensionless stroke volume from CMR measurements (MPG_CMR-Predicted_).

A new predictive model based on vorticity and dimensionless oscillating flow (Strouhal number) was evaluated and led to a better MPG estimation from CMR (Figure 2, panel C and D). The new MPG_CMR-Predited_ model introduced in this study showed that MPG can be accurately estimated by CMR and allowed to support the use of CMR as corroboration imaging technique for AS severity [3,4,8]. It may be also useful in the management of inconsistent severity grading (Figure 3), a current scenario between TTE and cardiac catheterization [15], and as it is demonstrated in this study (Table 2, Figure 3A), between TTE and CMR. Inconsistencies on EOA and MPG measurements may lead to incorrect therapeutic/surgical decisions. It is important to avoid measurement inconsistencies as reported with cardiac catheterization and define consistent cut-offs (EOA and pressure gradients) valid on all imaging techniques used to assess AS severity.

Furthermore, energy loss was evaluated using CMR and was strongly associated to MPG_error_, mean vorticity intensity and dimensionless oscillating flow. A recent substudy of SEAS cohort [22] showed the potential usefulness of energy loss, pressure recovery and energy loss coefficient ([EOA×A_Ao_] / [A_Ao_-EOA]) for AS severity assessment, highlighting the importance of this parameter unexplored in CMR. A more accurate evaluation of energy loss and vorticity may be computed using CMR 4D flow velocity measurements [29,38-40].

The cohort and results presented in this study make part of an ongoing prospective study at our institution. Some of our previous works included a part or the integrity of the actual cohort exploring different topics. Briefly, our work comparing continuity equation EOA using TTE and CMR used 48% of the actual data [3], the introduction a novel EOA method with CMR used 57% [5], two closely related works exploring pressure gradient difference between TTE and CMR used 48% [16], and the same cohort [17]. Finally, the cohort presented in this work was included in a more recent study evaluating the valve EOA kinetic in patients with AS [6]. Patients with bicuspid valve showed a ratio close to 30% of the ongoing population.

In terms of methodology it is important to observe that the initial imaging work flow included measurements at 6 mm and 10 mm downstream from the aortic valve. Measurements at 6 mm showed to be slightly higher than those at 10 mm [3]. However, 6 mm plane is unusual in clinical practice [4] and it was found none statistical difference between both velocity planes. So our work flow was modified in consequence. Parallel plane position to the aortic valve annulus may crucial for the adequate measurement of velocity gradients at vena contracta position, avoiding potential sources of velocity underestimation [4,5,8]. In particular the presence of high eccentric flow jets, often observed in patients with bicuspid aortic valves and aortic dilation, may contribute to pressure gradient underestimation [41,42]. However, AS severity does not seem to be the only factor affecting flow jet eccentricity, complex rotatory flow patterns and velocity-related measurements [42,43]. In this study non-statistical difference was found when comparing bicuspid vs. tricuspid MPG.

Vortex formation is currently visually evaluated for identifying abnormal flow patterns [41,42,44,45]. However vorticity intensity provides a quantitative approach of vorticity and vortex formation. In this study MPG, EOA, energy loss, and Strouhal number (dimensionless oscillating flow) were significantly associated with mean vorticity. Vorticity was also used to compute EOA using vorticity jet shear layer detection method [5,17,29], this method may be more accurate than continuity equation and may be useful for differentiating pseudo-severe AS to truly-severe AS severity at rest or during dobutamine perfusion [23] given its high-accurate fluid mechanics approach, non-circular LVOT shape assumptions and/or stroke volume computation need. Vorticity intensity may provide useful additional information of aortic valve hemodynamics and AS severity.

### Study limitations

Accurate estimation of valve vorticity magnitude is dependent on the temporal and spatial resolution (typically 30–40 ms per phase and 1–2 mm, respectively) which is essentially determined by patient’s heart rate, sequence design and echo time. In this study we use a vendor product flow sequence which automatically estimates the shorter TE, however TE was maybe not enough shorter to accurately measure peak velocities [14]. New promising fast acquisition flow sequences and hardware (i.e. SENSE, image mapping, ultra-short echo time, and parallel imaging) could help overcoming these limitations. It is important to notice that vorticity was calculated using standard through-plane velocity measurements (i.e. a single velocity component). As presented in the discussion full velocity vector acquisition may provide a more accurate estimation of vorticity. The number of patients with AS and adverse events was too small (7 valve replacement surgeries) to determine the association between valve vorticity intensity with clinical outcomes.

## Conclusion

In conclusion, this study showed that flow vorticity is one of the main factors responsible for the MPG discrepancies between CMR and TTE. It is possible to account for this factor and correct the MPG measured by CMR. Larger studies are needed to confirm the potential usefulness of CMR-derived vorticity and flow-derived parameters in cardiovascular diseases and valve function.

## Competing interests

The authors declared that they have no competing interests.

## Authors’ contributions

All authors contributed to the scope and outline of the manuscript. JG wrote the final draft. All authors read and approved the final manuscript.
